# Adjuvant Therapy With PD1/PDL1 Inhibitors for Human Cancers: A Systematic Review and Meta-Analysis

**DOI:** 10.3389/fonc.2022.732814

**Published:** 2022-02-25

**Authors:** Yao Jin, Jiayan Wei, Yiming Weng, Jia Feng, Zexi Xu, Peiwei Wang, Xue Cui, Xinyi Chen, Jinsong Wang, Min Peng

**Affiliations:** Department of Oncology, Renmin Hospital of Wuhan University, Wuhan, China

**Keywords:** human cancers, immune checkpoint inhibitor, PD1, PDL1, adjuvant therapy, meta-analysis

## Abstract

**Background:**

Immune checkpoint inhibitors (ICIs) have made a breakthrough in the systemic treatment of patients with advanced tumors. However, little is known about their efficacy and safety in adjuvant settings after the resection of solid tumors.

**Methods:**

We performed a meta-analysis on the efficacy and safety of programmed death 1 (PD1)/PD-1 ligand (PDL1) inhibitors in adjuvant therapy after tumor resection using Review Manager 5.3, based on published clinical studies. The outcomes included recurrence-free survival (RFS), disease-free survival (DFS), overall survival (OS), and adverse events (AEs).

**Results:**

Eight randomized controlled trials (RCTs) were included in the analysis. The use of PD1/PDL1 inhibitors in adjuvant therapy significantly improved RFS (hazard ratio [HR] = 0.72; 95% confidence interval [CI] 0.67–0.78, p < 0.00001). However, there was no statistically significant difference in OS between PD1/PDL1 inhibitors and placebo (HR = 0.86; 95% CI 0.74–1.00, p = 0.05). Gender, age, and PDL1 status were independent predictors of RFS with PD1/PDL1 inhibitors. As for the safety analysis results, PD1/PDL1 inhibitors had a higher incidence of fatigue (risk ratio [RR] = 1.22; 95% CI 1.01–1.49, p = 0.04), nausea (RR = 1.47; 95% CI 1.11–1.94, p = 0.007), and pruritus (RR = 1.96; 95% CI 1.57–2.44, p < 0.00001). In addition, the incidence of any grade adverse events increased in the PD1/PDL1 inhibitor group (RR = 1.03; 95% CI 1.02–1.05, p < 0.0001).

**Conclusions:**

This is the first meta-analysis on the efficacy and safety of PD1/PDL1 inhibitors in adjuvant therapy. The use of PD1/PDL1 inhibitors in adjuvant therapy could significantly reduce the recurrence rate after solid tumor resection. However, the incidence of fatigue, nausea, pruritus, and any grade AEs also increased, which should be monitored with vigilance.

## 1 Introduction

As per the theme of ASCO 2021, adjuvant therapy is currently the most popular research direction in oncology. Subgroups of patients with high-risk characteristics of primary tumor and regional lymph node metastasis are at an increased risk of recurrence and a poor prognosis after surgical resection (1, 2). The aim of adjuvant therapy is to eliminate minimal residual disease (MRD) after resection (3, 4). Moreover, it has been found in a variety of solid tumors to improve recurrence-free survival and improve overall survival (5–8). However, in some studies, systemic adjuvant therapy did not provide a significant survival benefit for cancer patients. For example, the STORM trial found that sorafenib in adjuvant therapy for liver cancer patients not only failed to bring survival benefits but also increased the risk of side effects and even death (9). A review by Alessandro et al. revealed that the efficacy of systemic adjuvant therapy for resected biliary tract cancer remains controversial (10). From this perspective, the choice of adjuvant therapy for cancer patients needs to be further explored.

Immune checkpoint inhibitors, such as nivolumab and pembrolizumab, have greatly changed the treatment pattern of several types of advanced and metastatic solid tumors in the past decade, and most importantly, they have achieved significant results (11–13). The results of the ASCO-PACIFIC study in 2021 showed that durvalumab was associated with a higher 5-year recurrence-free survival (RFS) (HR = 0.55; 95% CI 0.45–0.68) and 5-year OS (HR = 0.72; 95% CI, 0.59–0.89) compared to the placebo group in patients after concurrent chemoradiation with unresectable stage III non-small cell lung cancer (NSCLC). All the above evidence indicates that immune checkpoint inhibitors (ICIs) have great potential as an adjuvant therapy strategy for high-risk recurrence tumors. Currently, many large-scale randomized controlled trials (RCTs) (14–18) have focused on the role of ICIs in adjuvant therapy. Some studies have revealed the superiority of ICI as an adjuvant therapy for cancer patients, while others indicated the opposite. Therefore, based on all the clinical study data published thus far, including the latest results of the 2021 ASCO conference, we conducted a systematic review and meta-analysis to provide a higher level of evidence-based medical recommendations on this clinical issue.

## 2 Materials and Methods

### 2.1 Search Strategy and Study Selection

A systematic literature search was conducted using the following databases: EMBASE, MEDLINE (PubMed), and Web of Science to identify eligible articles published before June 2021. The search terms mainly included adjuvant therapy, PD1 inhibitors, PDL1 inhibitors, immune checkpoints, and cancer. Details of the retrieval strategy are provided in the **Supplementary Material**.

The included studies were selected based on the following criteria (1): study type: randomized controlled trials (RCTs) (2); participants: patients with solid tumors that were histologically confirmed (3); experimental group: PD1/PDL1 inhibitors were used in adjuvant therapy after surgical resection; control group: placebo or drugs other than PD1/PDL1 inhibitors were used in adjuvant therapy; and (4) outcomes: overall survival (OS), recurrence-free survival (RFS), disease-free survival (DFS), and drug safety. The exclusion criteria were as follows (1): the number of patients <20, non-RCTs (2), insufficient data to estimate, and (3) non-English translation.

### 2.2 Data Extraction

We extracted the following information from each study: name of first author, year of publication, type of tumor, phase of trials, experimental group and control group, number of patients, hazard ratios (HRs) and confidence intervals (CIs) for outcomes (OS, RFS, and DFS), and the number of patients with adverse events.

### 2.3 Quality Assessment

Review Manager 5.3 was used to evaluate the quality of the included studies. The evaluation items included random sequence generation, allocation concealment, blinding of participants and personnel, blinding of outcome assessment, incomplete outcome data, selective reporting, and other sources of bias. Each item was evaluated and resulted as being either high risk, low risk, or unclear.

### 2.4 Statistical Analysis

Review Manager 5.3 was used to analyze the data. In this meta-analysis, HRs and their 95% CIs for outcomes (RFS, DFS, and OS) were used to calculate the pooled results. For dichotomous outcomes, the number of events and total patients in the experimental and control groups were extracted and used to calculate the risk ratio (RR). Differences were considered statistically significant at p < 0.05. The I^2^ statistic was used to evaluate the heterogeneity across the included studies. If I^2^ > 50%, it was considered that there was significant heterogeneity across the studies, and the random-effects model was selected. Otherwise, the fixed-effects model was selected. The source of heterogeneity was analyzed using subgroup and sensitivity analyses. Publication bias was assessed using funnel plots.

## 3 Results

### 3.1 Study Characteristics

**Figure 1:**
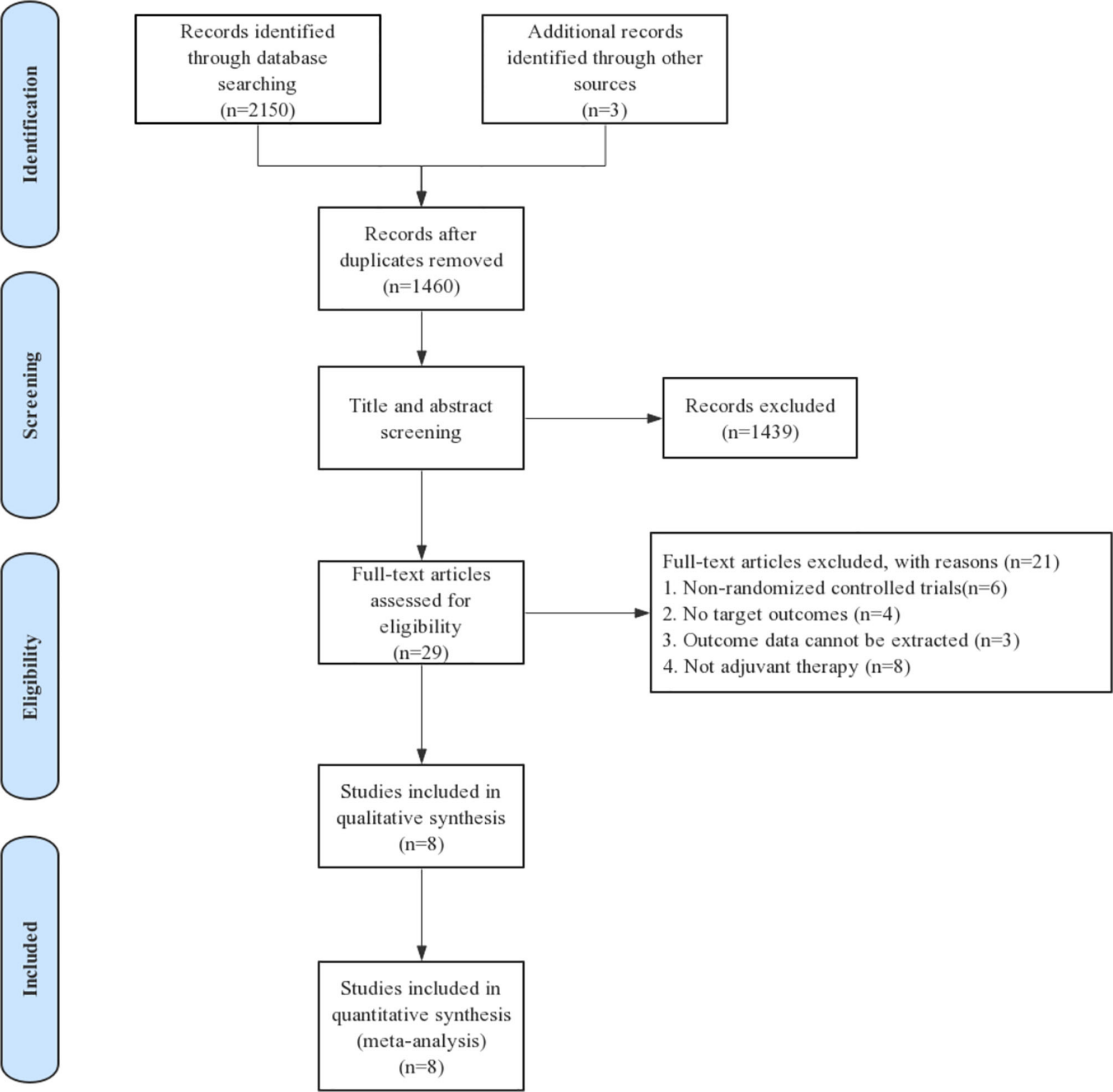
PRISMA flow diagram the search strategy and results.

Eligible studies were identified and selected as shown in **Figure 1**. In total, 2,153 articles were initially evaluated, and 1,460 studies were eligible after exclusion of duplicates. The abstracts and titles of these studies were reviewed, and 1,439 studies were excluded. After an abstract review, we identified 29 articles for full manuscript review, and 21 of these articles were excluded for the reasons delineated in **Figure 1**. Finally, eight RCTs involving more than 6,000 patients were included in our study. Of the tumor types, three studies were conducted on melanoma, one study on esophageal cancer or gastroesophageal junction cancer, one study on NSCLC, one study on renal cell carcinoma, and two studies on urothelial carcinoma. The characteristics of each study are summarized in **Table 1**.

**Table 1:** Characteristics of the included studies.

Authors	Year	Cancer type	RCT phase	PD1/PDL1 inhibitor	Control group	Case	HR (CI) for PD1/PDL1 inhibitor	
							RFS	OS
Ascierto et al.	2020	Melanoma	3	Niv 3 mg/kg Q2W	Ipi 10 mg/kg Q3W	906	0.71 (95% CI 0.60–0.86)	0.87 (95% CI 0.66–1.14)
Bellmunt et al.	2021	Urothelial carcinoma	3	Ate 1,200 mg Q3W	Observation	809	0.89 (95% CI 0.74–1.08)	0.85 (95% CI 0.66–1.09)
Eggermont et al.	2018	Melanoma	3	Ate 1,200 mg Q3W	Placebo Q3W	1019	0.57 (98.4% CI 0.43–0.74)	–
Kelly et al.	2021	Esophageal or gastroesophageal junction cancer	3	Niv 240 mg Q2W	Placebo Q2W	794	0.69 (96.4% CI 0.56–0.86)	–
Zimmer et al.	2020	Melanoma	2	Niv 3 mg/kg Q2W	Placebo Q2W	111	0.56 (97.5% CI 0.33–0.94)	–
Bajorin et al.	2021	Urothelial carcinoma	3	Niv 240 mg Q2W	Placebo Q2W	709	0.70 (98.31% CI 0.54–0.89)	–
Wakelee et al.	2021	Non-small cell lung cancer	3	Niv 240 mg Q2W	Best standard care	1005	0.79 (95% CI 0.64–0.96)	0.99 (95% CI 0.73–1.33)
Choueiri et al.	2021	Renal cell carcinoma	3	Pem 200 mg Q3W	Placebo Q3W	994	0.68 (95% CI 0.53–0.87)	0.54 (95% CI 0.30–0.96)

Ate, atezolizumab; Ipi, ipilimumab; Niv, nivolumab; Pem, pembrolizumab; HR, hazard ratio; PD1, programmed death 1; PDL1, programmed death 1 ligand; RFS, recurrence-free survival; OS, overall survival; Q2W, every 2 weeks; Q3W, every 3 weeks; RCT, randomized controlled trials.

### 3.2 Risk of Bias

**Figure 2:**
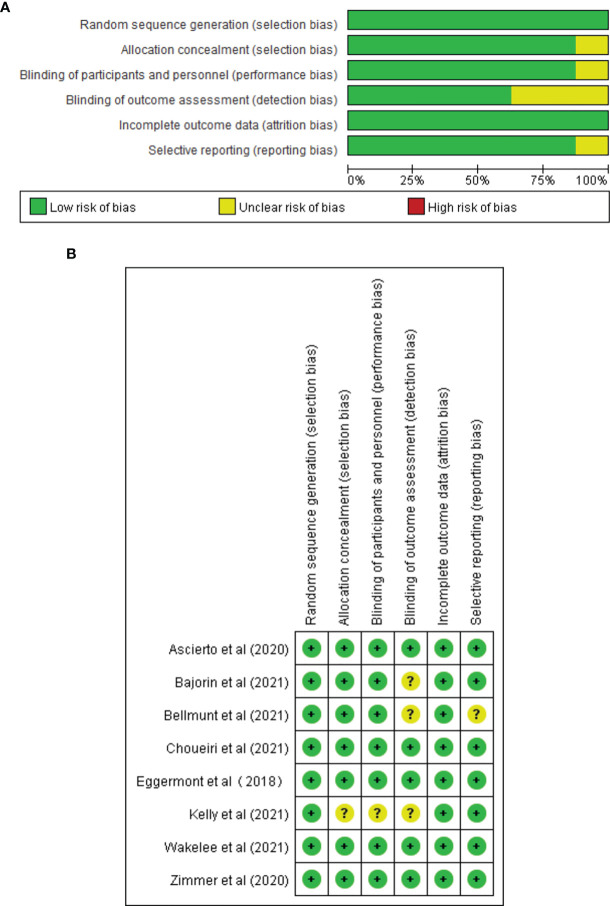
Risk of bias assessment at the study level. **(A)** Risk of bias graph: review authors’ judgment about each risk of bias item presented as percentages across all included full reported studies. **(B)** Risk of bias summary: review authors’ judgment about each risk of bias item for each included study.

All included studies were RCTs; therefore, the overall risk of bias was relatively low. The quality evaluation results of the included studies are shown in **Figures 2A** **, B**.

### 3.3 Analysis of Efficacy Outcomes

#### 3.3.1 Recurrence-Free Survival

**Figure 3:**
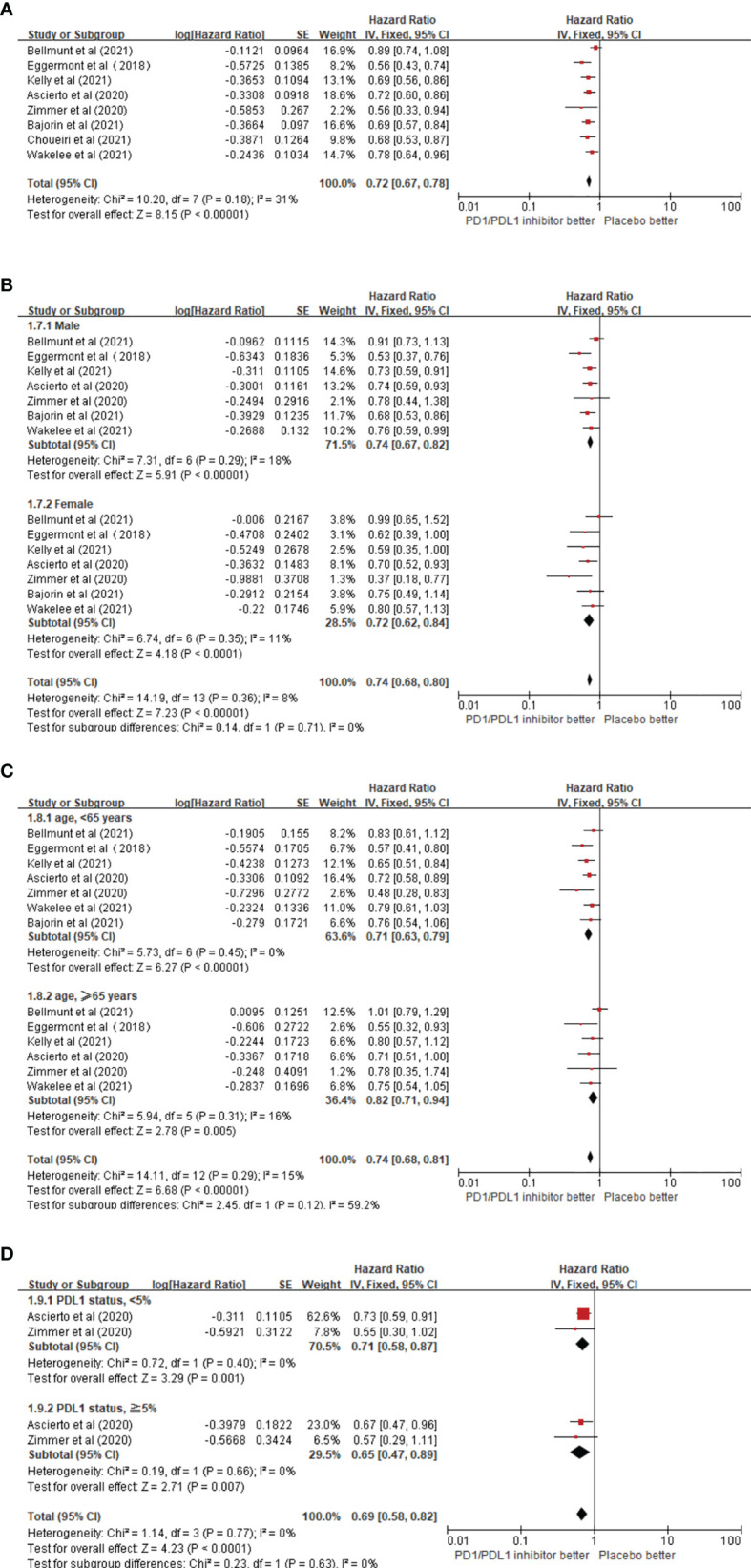
**(A)** Forest plots of the fixed-effects meta-analysis for the effects of PD1/PDL1 inhibitors on RFS. **(B)** Forest plots of the fixed-effects meta-analysis for the effects of PD1/PDL1 inhibitors on PFS in gender. **(C)** Forest plots of the fixed-effects meta-analysis for the effects of PD1/PDL1 inhibitors on RFS in different age group. **(D)** Forest plots of the fixed-effects meta-analysis for the effects of PD1/PDL1 inhibitors on RFS in different PDL1 status.

Overall, eight trials on the RFS of patients receiving ICIs in adjuvant therapy involving 6,347 patients were reviewed. The pooled results revealed that the use of PD1/PDL1 inhibitors in adjuvant immunotherapy can significantly reduce the risk of recurrence after tumor resection (HR = 0.72; 95% CI 0.67–0.78, p < 0.00001) (**Figure 3A**). The study by Bellmunt et al. was a source of heterogeneity (I^2^ = 31%), in which atezolizumab was the experimental arm. The source of heterogeneity could be that it was the only study in which the experimental group was a PDL1 inhibitor. In the gender subgroup analysis, both men and women could obtain RFS benefits from adjuvant therapy with PD1/PDL1 inhibitors. HR was 0.74 (95% CI 0.67–0.82, p < 0.00001) and 0.72 (95% CI 0.62–0.84, p < 0.0001), respectively (**Figure 3B**). In the age subgroup analysis, longer RFS could be obtained from the adjuvant treatment of PD1/PDL1 inhibitors for those aged <65 years (HR = 0.71; 95% CI 0.63–0.79, p < 0.00001) or older than 65 years (HR = 0.82; 95% CI 0.71–0.94, p = 0.005) (**Figure 3C**). In the PDL1 status subgroup analysis, the use of PD1/PDL1 inhibitors in adjuvant therapy compared with placebo reduced the risk of disease recurrence in the subgroups with <5% or ≥5% PDL1 status (**Figure 3D**).

#### 3.3.2 Overall Survival

**Figure 4:**
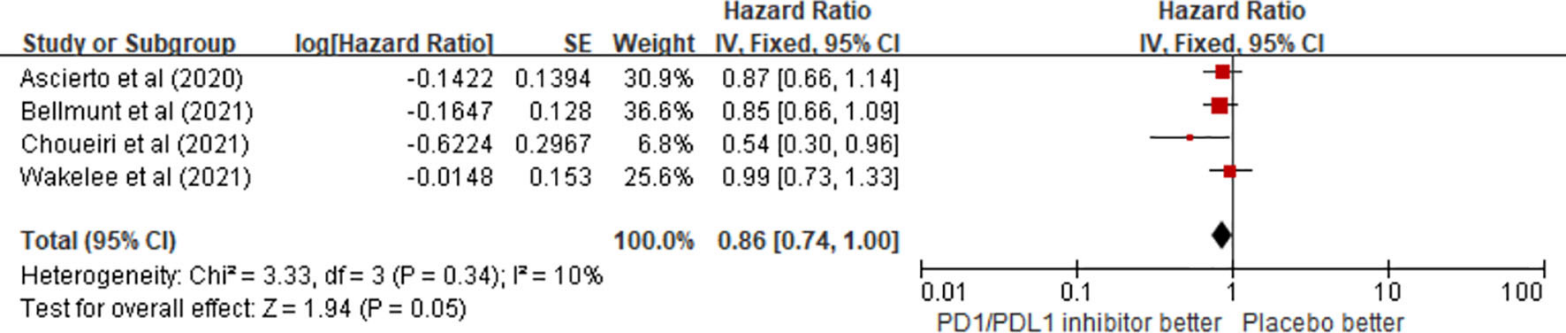
Forest plots of the fixed-effects meta-analysis for the effects of PD1/PDL1 inhibitors on OS.

Regarding OS benefits, a total of four trials on the OS of patients receiving ICIs as adjuvant therapy involving 3,714 patients were reviewed. The pooled results showed that there was no statistical difference in OS benefit between the PD1/PDL1 inhibitor arm and the placebo arm in adjuvant therapy (HR = 0.86; 95% CI 0.74–1.00, p = 0.05) (**Figure 4**).

### 3.4 Analysis of Safety Outcomes

#### 3.4.1 Any Grade Adverse Events

**Figure 5:**
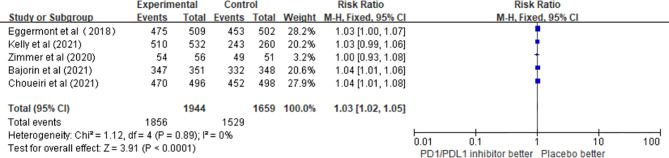
The risk of any grade AEs in the PD1/PDL1 inhibitors groups and placebo groups.

A total of five studies involving 3,603 patients confirmed the safety of PD1/PDL1 inhibitors. The pooled results revealed that the risk of any grade adverse events (AEs) was significantly higher in the adjuvant therapy with PD1/PDL1 inhibitors than in the control group (RR = 1.03; 95% CI 1.02–1.05, p < 0.0001) (**Figure 5**).

#### 3.4.2 Subgroup Analysis of Any Grade Adverse Event

**Figure 6:**
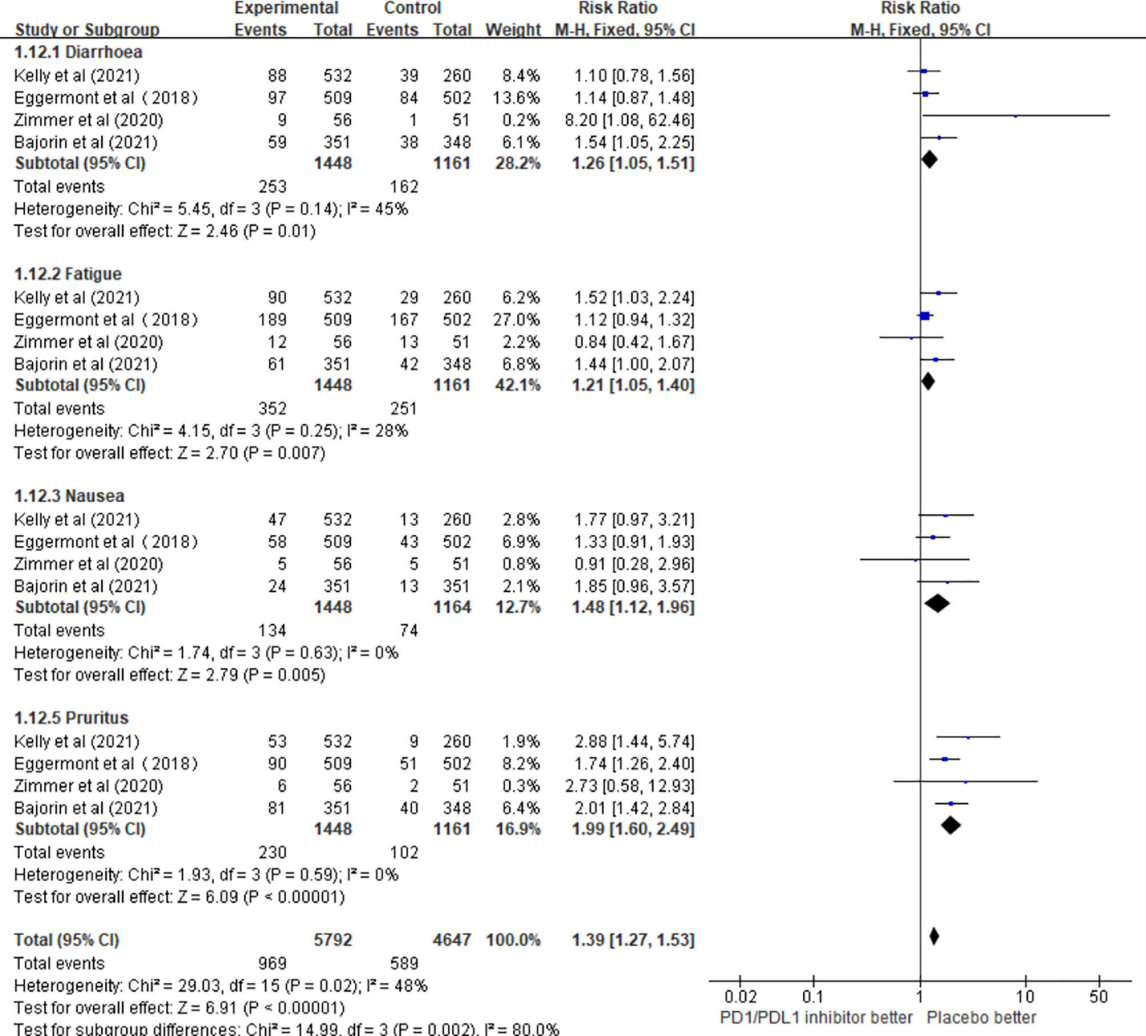
The incidence of different adverse events in the PD1/PDL1 inhibitors groups and placebo groups.

The results of this meta-analysis showed that the incidence of fatigue (RR = 1.22; 95% CI 1.01–1.49, p = 0.04), nausea (RR = 1.47; 95% CI 1.11–1.94, p = 0.007), and pruritus (RR = 1.96; 95% CI 1.57–2.44, p < 0.00001) in patients who received PD1/PDL1 inhibitors in adjuvant therapy was significantly higher than that in the control group. However, there was no significant difference in the incidence of diarrhea (RR = 1.27; 95% CI 0.96–1.68, p = 0.09) (**Figure 6**).

## 4 Publication Bias

**Figure 7:**
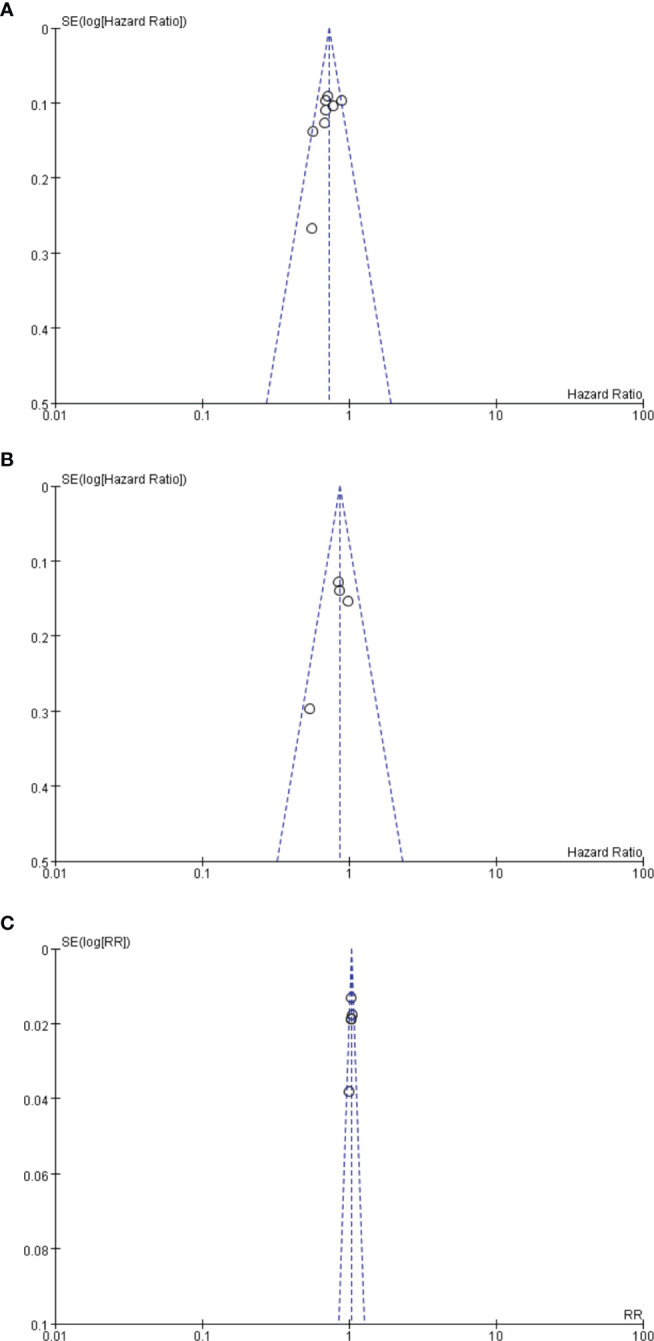
**(A)** Funnel plot analysis of potential publication bias for RFS. **(B)** Funnel plot analysis of potential publication bias for OS. **(C)** Funnel plot analysis of potential publication bias for any advent events.

Funnel plot analysis neither indicated apparent publication bias affecting the HRs for RFS and OS nor showed apparent publication bias on RRs of any adverse events (**Figure 7**).

## 5 Discussion

Immune checkpoint inhibitors have been widely used in patients with several types of advanced and metastatic solid tumors and have achieved significant OS benefits (19–21). At present, adjuvant therapy is the theme of the ASCO Conference in 2021 and has become the focus of the current oncology therapy field. Several large clinical studies have focused on the adjuvant therapy of ICIs, but the conclusions have been incongruent (22–24). To the best of our knowledge, this is the first systematic review and meta-analysis on the efficacy and safety of PD1/PDL1 inhibitors in the adjuvant treatment of solid tumors after solid tumor resection.

The pooled results of this meta-analysis showed that PD1/PDL1 inhibitors were effective as an adjuvant therapy for tumors. This is consistent with the conclusions of previous studies that explored the efficacy of CTLA-4 inhibitors in adjuvant therapy. The phase III EORTC 18071 trial (25, 26) demonstrated that ipilimumab significantly improved 3-year RFS (HR = 0.75; 95% CI 0.64–0.90, p = 0.0013) after complete resection of stage III melanoma compared to placebo. This study led to the approval of ipilimumab for stage III melanoma after resection in 2015 (27) and was the first immune checkpoint inhibitor approved for adjuvant therapy. In the NCT02523313 phase trial, patients with resected stage IV melanoma with no evidence of disease receiving nivolumab plus ipilimumab in adjuvant therapy had significantly longer RFS (HR = 0.23; 97.5% CI 0.12–0.45, p < 0.0001) than those in the placebo group (16).

Our pooled results also revealed that in the subgroup analysis, patients younger than and older than 65 years could benefit from PD1/PDL1 inhibitors. In patients older than 65 years, PD1/PDL1 inhibitors reduced the risk of recurrence by 18%, and a greater benefit was observed in patients younger than 65 years. This is inconsistent with the conclusion of the EORTC-18071 trial, which revealed that there was no significant difference between the ipilimumab and placebo groups in RFS benefits for patients older than 65 years of age (25). In patients with PDL1 status ≥5%, a 35% reduction in recurrence risk was observed in the PD1/PDL1 inhibitor arm, and comparable results were observed in patients with PDL1 < 5%. This is consistent with the conclusions of two previous clinical trials. In the NCT02362594 trial (28) confirming the efficacy of pembrolizumab in the adjuvant therapy of stage III melanoma and the phase III NCT02743494 trial (15) exploring the role of nivolumab in adjuvant therapy for esophageal or gastroesophageal junction cancer, RFS benefit was observed in patients receiving pembrolizumab or nivolumab regardless of whether PDL1 expression was >1% or ≤1%. However, this is inconsistent with the results of the IMvigor010 trial, which revealed that regardless of the expression status of PDL1, atezolizumab did not improve DFS compared with placebo (14). The tumor types involved in our meta-analysis included melanoma, urothelial carcinoma, renal cancer, NSCLC, and esophageal or gastroesophageal junction cancer. There are currently many ongoing clinical trials exploring the efficacy of ICIs in adjuvant therapy for various tumor types. For example, the NCT02196961 trial is ongoing to explore the efficacy of ipilimumab or nivolumab in the adjuvant therapy of Merkel cell carcinoma (27), and the efficacy and safety of pembrolizumab are being confirmed for stage III or IV melanoma after resection in the phase III clinical trials SWOG S1404 (3, 27).

In addition, our results showed that PD1/PDL1 inhibitors did not improve OS in adjuvant therapy, which might be explained by the following reasons. In the study by Ascierto et al. (17), both the experimental and control groups were ICIs (nivolumab versus ipilimumab). In addition, effective immunotherapy or targeted therapy was subsequently used, leading to possible inherent crossover. In the study by Bellmunt et al. (14), the OS data were not complete because it was still in follow-up, and the use of ICIs in the late control group may have affected the OS. However, in the 2021 ASCO-Pacific study, durvalumab significantly improved OS in patients with unresectable stage III NSCLC following concurrent chemoradiotherapy. The difference in OS benefit may be due to the difference in efficacy between concurrent chemoradiotherapy and tumor resection.

The results of this meta-analysis revealed an increased risk of any grade AEs, fatigue, nausea, and pruritus in adjuvant therapy with PD1/PDL1 inhibitors relative to placebo, which is consistent with the safety results in advanced and metastatic cancer patients receiving PD1/PDL1 inhibitors (29–32). Therefore, these findings should be noted during the use of PD1/PDL1 inhibitors in the adjuvant therapy of solid tumors.

Tumor cells can escape the immune system by activating the T-cell suppression pathway, which is the immune checkpoint pathway. One of the most important pathways is the PD1 pathway (33–35). PD1 is expressed on the surface of T cells in the tumor microenvironment and binds to two ligands (PDL1 and PDL2), resulting in inactivation of the T cells’ tumor-specific immune response, thus allowing the tumor to progress (36–38). PD1/PDL1 inhibitors are antagonists targeting PD1 or PDL1 sites. Therefore, the use of these two drugs will activate the immune response of T cells to tumors (39, 40), thereby inhibiting the growth of tumor cells. MRD is usually present after resection of solid tumors, which is the main cause of tumor recurrence (39, 41). Tumor load is greatly reduced after tumor resection; thus, immune cells are more likely to come into contact with the remaining tumor cells and kill them. PD1/PDL1 inhibitors have the potential to eliminate MRD and thus may reduce the risk of recurrence in patients after tumor resection. In addition, for patients that are in poor physical condition during the perioperative period, clinicians may opt to use an immunotherapy with a lower incidence of adverse events compared to radiotherapy or chemotherapy.

We performed the first meta-analysis of the efficacy and safety of PD1/PDL1 inhibitors in adjuvant therapy. Apart from nivolumab in melanoma, no ICIs have been approved for adjuvant therapy, and the results of our meta-analysis may provide evidence for new clinical applications of ICIs in the future and opens a new avenue for systemic adjuvant therapy. At present, the study content of this topic cannot be applied to clinical practice, which needs to be verified by many large randomized clinical trials in the future. In addition, clinical decision-making requires a reasonable balance between the efficacy and toxicity of PD1/PDL1 inhibitors in adjuvant therapy. For future research on this topic, we think that the following aspects can be expanded on. First, to better play the role of PD1/PDL1 inhibitors in adjuvant therapy, we need to select appropriate patients, namely, the applicable population. Second, the specific regimen and dose selection of PD1/PDL1 inhibitors in adjuvant therapy still need to be further explored. Third, the efficacy predictors of PD1/PDL1 inhibitors in adjuvant therapy for cancer patients need to be explored and updated, such as blood indicators, which have guiding significance for when to stop and whether to continue using drugs. Fourth, the application of PD1/PDL1 inhibitors in combination with other therapies such as targeted therapy or radiotherapy in adjuvant therapy may also be a new breakthrough point in the future.

Our meta-analysis has several limitations. First, many ongoing clinical trials have not yet been completed. Second, due to the diversity of cancer types and adjuvant treatment options and the limited number of included studies, we were unable to conduct a subgroup analysis on the various cancer types and treatment options. This was one of the sources of the heterogeneity in the study results. In the future, more studies on the use of PD1/PDL1 inhibitors in the adjuvant treatment of cancer patients will be conducted, and this will give the study greater statistical significance. Third, although the heterogeneity between the results of each analysis was not particularly significant, the study of Bellmunt et al. was the main source of heterogeneity after the heterogeneity test, which may be related to the PDL1 inhibitor in the experimental group. This suggests that there may be great heterogeneity between the efficacy and safety of PD1 and PDL1 inhibitors, and more studies are needed to confirm this.

## 6 Conclusion

Overall, the results of our meta-analysis revealed that the use of PD1/PDL1 inhibitors in adjuvant therapy was associated with better RFS compared to controls. Men or women older than or younger than 65 years of age can benefit from PD1/PDL1 inhibitors. Moreover, regardless of the expression status of PDL1, PD1/PDL1 inhibitors can reduce the risk of recurrence. However, the use of PD1/PDL1 inhibitors in adjuvant therapy also increases the risk of adverse events such as fatigue, nausea, and pruritus. Our results provide a reference for the application of PD1/PDL1 inhibitors as adjuvant therapy for solid tumors. However, more studies are needed to demonstrate the efficacy and safety of PD1/PDL1 inhibitors in adjuvant therapy.

## Data Availability Statement

The original contributions presented in the study are included in the article/**Supplementary Material**. Further inquiries can be directed to the corresponding author.

## Author Contributions

Conceptualization, YW, MP. Methodology, YJ, ZX. Software, JyW. Formal analysis, JsW, XyC. Writing—original draft preparation, YJ, JyW, PW, XC. Writing—review and editing, YW. All authors contributed to the article and approved the submitted version.

## Funding

This work was supported by grants from the National Natural Science Foundation of China (81770169), National Natural Science Foundation of China (81802980), and National Natural Science Foundation of China (81102024).

## Conflict of Interest

The authors declare that the research was conducted in the absence of any commercial or financial relationships that could be construed as a potential conflict of interest.

## Publisher’s Note

All claims expressed in this article are solely those of the authors and do not necessarily represent those of their affiliated organizations, or those of the publisher, the editors and the reviewers. Any product that may be evaluated in this article, or claim that may be made by its manufacturer, is not guaranteed or endorsed by the publisher.
