# Supplementary material for: Adjuvant Therapy With PD1/PDL1 Inhibitors for Human Cancers: A Systematic Review and Meta-Analysis
Source: Front Oncol. 2022 Feb 25;12:732814. doi: 10.3389/fonc.2022.732814 (PMC8913885; doi:10.3389/fonc.2022.732814)
Supplement: Supplementary file 1 [file DataSheet_1.docx]

**Literature search strategy.**

1. **Database: PubMed**

| **Search number** | **Query** | **Results** |
| --- | --- | --- |
| #6 | ((((immune checkpoint inhibitor) OR (PD1 inhibitor)) OR (PDL1 inhibitor)) AND (adjuvant therapy)) AND (("Neoplasms"[Mesh]) OR (((((((((((((((((Neoplasia) OR (Neoplasias)) OR (Neoplasm)) OR (Tumors)) OR (Tumor)) OR (Cancer)) OR (Cancers)) OR (Malignancy)) OR (Malignancies)) OR (malignant neoplasms)) OR (malignant neoplasm)) OR (neoplasm malignant)) OR (neoplasms malignant)) OR (benign neoplasms)) OR (neoplasms benign)) OR (benign neoplasm)) OR (neoplasm benign))) | 1,122 |
| #5 | ((immune checkpoint inhibitor) OR (PD1 inhibitor)) OR (PDL1 inhibitor) | 25,173 |
| #4 | adjuvant therapy | 237,119 |
| #3 | ("Neoplasms"[Mesh]) OR (((((((((((((((((Neoplasia) OR (Neoplasias)) OR (Neoplasm)) OR (Tumors)) OR (Tumor)) OR (Cancer)) OR (Cancers)) OR (Malignancy)) OR (Malignancies)) OR (malignant neoplasms)) OR (malignant neoplasm)) OR (neoplasm malignant)) OR (neoplasms malignant)) OR (benign neoplasms)) OR (neoplasms benign)) OR (benign neoplasm)) OR (neoplasm benign)) | 5,008,497 |
| #2 | ((((((((((((((((Neoplasia) OR (Neoplasias)) OR (Neoplasm)) OR (Tumors)) OR (Tumor)) OR (Cancer)) OR (Cancers)) OR (Malignancy)) OR (Malignancies)) OR (malignant neoplasms)) OR (malignant neoplasm)) OR (neoplasm malignant)) OR (neoplasms malignant)) OR (benign neoplasms)) OR (neoplasms benign)) OR (benign neoplasm)) OR (neoplasm benign) | 5,008,497 |
| #1 | "Neoplasms"[Mesh] | 3,489,622 |

1. **Database: Embase**

| **Search number** | **Query** | **Results** |
| --- | --- | --- |
| #22 | #19 AND #20AND #21 | 386 |
| #21 | 'adjuvant therapy' | 142744 |
| #20 | 'immune checkpoint inhibitor' OR 'pd1 inhibitor' OR 'pdl1 inhibitor' | 10341 |
| #19 | #1 OR #2 OR #3 OR #4 OR #5 OR #6 OR #7 OR #8 OR #9 OR #10 OR #11 OR #12 OR #13 OR #14 OR #15 OR #16 OR #17 OR #18 | 5363052 |
| #18 | 'neoplasm, benign':ab,ti | 60 |
| #17 | 'benign neoplasm':ab,ti | 2433 |
| #16 | 'neoplasms, benign':ab,ti | 147 |
| #15 | 'benign neoplasms':ab,ti | 0 |
| #14 | 'neoplasms, malignant':ab,ti | 79 |
| #13 | 'neoplasm, malignant':ab,ti | 62 |
| #12 | 'malignant neoplasm':ab,ti | 0 |
| #11 | 'malignant neoplasms':ab,ti | 1 |
| #10 | 'malignancies':ab,ti | 196675 |
| #9 | 'malignancy':ab,ti | 237568 |
| #8 | 'cancers':ab,ti | 410066 |
| #7 | 'cancer':ab,ti | 2606439 |
| #6 | 'tumor':ab,ti | 1691261 |
| #5 | 'tumors':ab,ti | 868977 |
| #4 | 'neoplasm':ab,ti | 85383 |
| #3 | 'neoplasias':ab,ti | 8062 |
| #2 | 'neoplasia':ab,ti | 78134 |
| #1 | 'malignant neoplasm'/exp | 3970179 |

1. **Database: Web of science**

| **Search number** | **Query** | **Results** |
| --- | --- | --- |
| #4 | #3 AND #2 AND #1 | 642 |
| #3 | TS= (adjuvant therapy) | 84863 |
| #2 | TS= (immune checkpoint inhibitor OR PD1 inhibitor OR PDL1 inhibitor) | 17494 |
| #1 | TS= (Neoplasia OR Neoplasias OR Neoplasm OR Tumors OR Tumor OR Cancer OR Cancers OR Malignancy OR Malignancies OR Malignant Neoplasms OR Malignant Neoplasm OR Neoplasm, Malignant OR Neoplasms, Malignant OR Benign Neoplasms OR Neoplasms, Benign OR Benign Neoplasm OR Neoplasm, Benign) | 3713660 |
